# Arbuscular Mycorrhizal Fungi and *Azospirillum brasilense* Enhance Growth and Resource Use Efficiency of Tomato Under Resource‐Limited Peat‐Reduced Cultivation

**DOI:** 10.1002/pei3.70205

**Published:** 2026-08-23

**Authors:** Ruvini Ranasingha, Aditya Prathipati

**Affiliations:** ^1^ School of Agriculture, Animal and Environmental Sciences Anglia Ruskin University Writtle Chelmsford UK

**Keywords:** arbuscular mycorrhizal fungi, *Azospirillum brasilense*, peat‐reduced substrates, sustainable horticulture, tomato ( *Solanum lycopersicum* )

## Abstract

The transition towards peat‐reduced and resource‐efficient horticultural systems presents challenges for maintaining crop productivity because of altered nutrient dynamics and reduced microbial diversity within soilless substrates. Beneficial microbial inoculants may provide a sustainable approach to improve plant performance under reduced‐input cultivation; however, their effectiveness in peat‐reduced hydroponic environments remains poorly understood. This study evaluated the effects of arbuscular mycorrhizal fungi (AMF; *Rhizophagus irregularis*) and nitrogen‐fixing bacteria (NFB; *Azospirillum brasilense* ), applied individually and in combination, on growth, productivity, resource‐use efficiency, and substrate nutrient dynamics of tomato grown under restricted fertigation in a peat‐reduced hydroponic system. NFB primarily enhanced vegetative growth, increasing leaf number by 11.7% and shoot fresh weight by 40%. AMF increased total fruit weight by 46% and exerted a significant positive main effect on WUE and NUE. Co‐inoculation produced the highest numerical responses, increasing shoot and root fresh biomass by 48% and 74%, fruit number by 80%, fruit weight by 77% and co‐inoculation increased substrate nitrate (NO_3_^−^) and potassium (K^+^) concentrations by 45% and 21%, respectively, compared with the control. Although co‐inoculation frequently produced the greatest numerical responses, interaction effects were generally limited, indicating complementary rather than strongly synergistic functions between the two microbial groups. Fruit quality characteristics, including total soluble solids and marketable fruit percentage, were not significantly affected by microbial treatments. Overall, these findings demonstrate complementary roles of AMF and *A. brasilens* under peat‐reduced cultivation, highlighting the potential of microbial inoculants to support more sustainable, low‐input greenhouse production systems.

## Graphical abstract

Microbial inoculation improved tomato performance in peat‐reduced hydroponic cultivation. *Azospirillum brasilense* primarily enhanced vegetative growth, arbuscular mycorrhizal fungi improved resource‐use efficiency, and co‐inoculation produced the highest numerical yield, demonstrating complementary rather than strongly synergistic microbial functions.

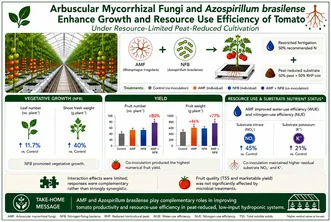

## 1 Introduction

*Solanum lycopersicum* L. (tomato) remains a cornerstone of global horticulture, valued for its substantial economic contribution and nutritional profile, including high levels of lycopene, vitamin C, and potassium (Rouphael et al. 2020; Włodarczyk et al. 2023). Commercial tomato production increasingly relies on greenhouse‐based soilless systems that enable precise management of water and nutrient supply (Lee and Lee 2015; D'Amico et al. 2023). These hydroponic and soilless systems effectively mitigate phytopathogen risks and enhance productivity (Sambo et al. 2019). However, many commercial operations continue to use peat‐based media due to their reliable moisture retention and nutrient buffering qualities, while others are actively developing and adopting peat‐reduced or peat‐free alternatives in response to environmental concerns and policy pressures (Maher et al. 2008; Aurdal 2025).

Peatlands represent major terrestrial carbon reservoirs, and their degradation through extraction, drainage, and land‐use change can release substantial quantities of stored carbon into the atmosphere, contributing to greenhouse gas emissions and climate change (Bednar‐Friedl et al. 2022; Billett et al. 2010). Consequently, environmental concerns and regulatory initiatives in many regions have accelerated the transition towards peat‐free and peat‐reduced growing media (Alexander et al. 2008; Aurdal 2025). Alternative substrates such as coir, wood fiber composted bark, and green waste compost are increasingly incorporated into commercial horticultural systems to reduce dependence on peat while maintaining suitable growing conditions (Schmilewski 2017; Maher et al. 2008).

Despite their environmental benefits, inert or low‐organic peat‐reduced substrates may create environments with reduced indigenous microbial diversity compared with conventional soils, which can affect nutrient uptake, stress tolerance, and resilience (Atzori et al. 2021; Trivedi et al. 2020; Müller et al. 2025). Reduced microbial activity in these substrates limits natural nutrient cycling, resulting in low nitrogen availability due to immobilization in high carbon‐to‐nitrogen materials such as wood fiber (Prasad et al. 2024; Wu et al. 2025). Consequently, greater reliance on external nutrient inputs may be required to maintain plant performance (Prasad et al. 2024). Such simplified microbial environments may also influence the establishment and persistence of introduced beneficial microorganisms within the rhizosphere. These challenges are further intensified by increasing pressure to reduce fertilizer and irrigation inputs while maintaining crop productivity, highlighting the need for strategies that improve resource‐use efficiency in soilless cultivation systems (Bednar‐Friedl et al. 2022; Zhang et al. 2015).

Microbial biostimulants have gained increasing attention in soilless and peat‐reduced cultivation systems as tools to restore biological activity, improve nutrient use efficiency, and enhance plant resilience (Ranasingha et al. 2024, 2025, 2026; Nie et al. 2025; Mannino 2025). Plant growth‐promoting rhizobacteria (PGPR) and nitrogen‐fixing bacteria (NFB) such as *Azospirillum brasilense* contribute to nitrogen fixation and stimulate root development under nutrient‐limited conditions (Ahemad and Kibret 2014; Ganusova et al. 2025). Arbuscular mycorrhizal fungi (AMF) establish symbiotic associations with plant roots, extending extraradical hyphal networks that enhance acquisition of relatively immobile nutrients, particularly phosphorus, while improving water relations and multiple physiological mechanisms (Slimani et al. 2022; Abarca et al. 2024; Othman et al. 2024). Evidence suggests that co‐inoculation of AMF and NFB can improve plant growth, nutrient acquisition, and stress tolerance, particularly under nutrient‐ or water‐limited conditions (Hnini et al. 2024; Shi et al. 2026).

Tomato‐specific studies demonstrate that microbial inoculation enhances growth, yield, physiological performance, and stress tolerance (Slimani et al. 2022; Mbogning et al. 2024). AMF and NFB have been associated with improved nutrient acquisition, plant growth, and stress tolerance in tomato (Abarca et al. 2024; Ullah et al. 2025). The soil‐based, open‐field study conducted by Bernados et al. (2024) demonstrated that co‐inoculation of AMF and *Azospirillum* spp. increased nutrient uptake and fruit yield in tomato, even under reduced chemical fertilizer, indicating synergistic effects of microbial inoculants. However, most available evidence has been generated under field or conventionally managed systems rather than peat‐reduced hydroponic environments.

Consequently, the effectiveness of microbial inoculants in peat‐reduced soilless systems operating under restricted water and nutrient inputs remains poorly understood. The present study therefore evaluated the individual and combined application of AMF and NFB in a peat‐reduced substrate under limited fertigation conditions. Specifically, the study investigated whether co‐inoculation with AMF and *A. brasilense* could enhance beneficial rhizosphere functions, improve nutrient acquisition and resource‐use efficiency, and sustain tomato productivity under reduced‐input cultivation conditions.

## 2 Materials and Methods

### 2.1 Experimental Site and Microclimatic Condition

**FIGURE 1:**
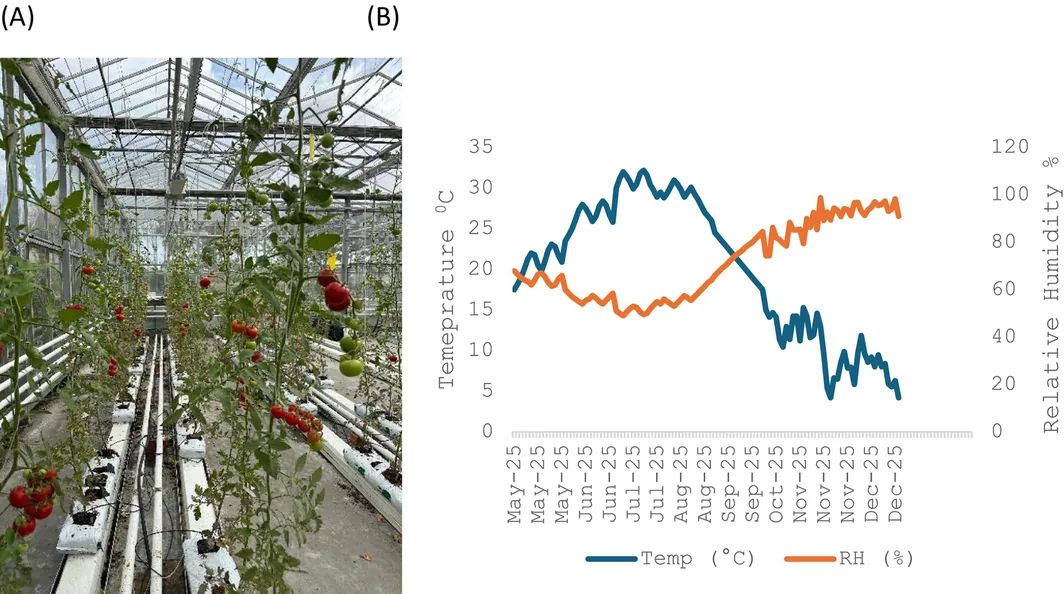
(A) Tomato plants grown in a glasshouse using grow bags and a drip irrigation system (photograph by the authors). (B) Environmental conditions inside the glasshouse during the study period, showing average daily temperature and relative humidity.

The research was conducted at the glasshouse facilities of ARU Writtle (Figure 1A), United Kingdom (51°43′24.4″N, 0°28′58.0″ E) from May to December 2025. Plants were grown under semi‐controlled glasshouse conditions under natural daylight, with PAR levels ranging between 300 and 600 μmol m^−2^ s^−1^. Temperature and relative humidity were recorded at 15‐min intervals using data loggers positioned throughout the glasshouse (Figure 1B).

### 2.2 Plant Material, Experimental Design and Microbial Inoculation

The selected planting material was *Solanum lycopersicum* L. cv. ‘Money Maker’, a widely used indeterminate cultivar with reliable performance under glasshouse conditions (Chira et al. 2024). Seeds were germinated in peat‐free compost and transplanted after 4 weeks into 5 kg peat‐reduced Jiffy Growbags containing 50% RHP‐certified coir and 50% peat. This substrate was classified as a peat‐reduced growing medium because peat was partially replaced with coir, in contrast to peat‐free substrates, which contain no peat. The substrate exhibited a pH of 5.5–6.0 and low electrical conductivity, providing suitable conditions for soilless cultivation.

The experiment was arranged as a 2 × 2 factorial randomized complete block design (RCBD) with five blocks and four treatments: control, arbuscular mycorrhizal fungi (AMF), nitrogen‐fixing bacteria (NFB), and AMF + NFB co‐inoculation. Each treatment was represented by three replicate grow bags per block, each containing six plants, resulting in a total of 360 plants. Treatments were randomly assigned within each block, and replicate grow bag was considered the experimental unit, resulting in 15 replicate grow bags (*n* = 15) per treatment.

Microbial inoculation employed *Rhizophagus irregularis* (AMF) and *Azospirillum brasilense* (NFB). Control plants received 200 mL water without inoculation. AMF was applied at transplanting as a root‐zone drench using DYNOMYCO (Groundwork BioAg) at 1.5 g L^−1^ (200 mL plant^−1^), delivering approximately 100–150 propagules per plant (Roussis et al. 2022; Slimani et al. 2022). NFB inoculation consisted of Contribute ibN containing *A. brasilense* CECT 9381 (1 × 10^9^ CFU g^−1^) applied at 2 g L^−1^ (200 mL plant^−1^) at transplanting and again at Week 2. The combined treatment received both inoculants at transplanting, followed by a second NFB application at Week 2.

### 2.3 Fertigation and Resource‐Limited Management

A reduced fertigation regime was implemented using Universol Violet (10–10–30) and Universol Blue (18–11–18) fertilizers. Stock solutions were prepared at 100 g L^−1^ and injected into the irrigation system at a dilution ratio of 1:200 using a Dosatron injector (Dosatron International, France), resulting in a final nutrient concentration of approximately 0.5 g L^−1^ (equivalent to 50–90 mg L^−1^ N, depending on fertilizer type). Nutrients were supplied through a drip fertigation system. Irrigation was applied three times daily, providing approximately 0.6 L plant^−1^ day^−1^ during the vegetative growth stage and 1.2 L plant^−1^ day^−1^ during flowering and fruit development. This regime was maintained throughout the experimental period to impose reduced‐input cultivation conditions in the peat‐reduced substrate.

### 2.4 Growth, Yield and Resource‐Use Efficiency Measurements

Vegetative growth and physiological responses were assessed bi‐weekly by measuring plant height, stem diameter, leaf number, flower number, chlorophyll content (SPAD‐502 m), and stomatal conductance (AP4 porometer) (Chaimala et al. 2023). At final harvest, shoot fresh weight (SFW), root fresh weight (RFW), shoot dry weight (SDW), and root dry weight (RDW) were determined. Biomass allocation was assessed using fresh and dry shoot‐to‐root ratios, while shoot and root dry matter percentages were calculated as (dry weight/fresh weight) × 100.

Fruit number was recorded from Week 3, and harvesting commenced at Week 6. Total fruit yield was determined as cumulative fruit fresh weight per plant. Marketable fruits (excluding cracked, misshapen, or undersized fruits) were quantified separately, and marketable fruit percentage was calculated as (marketable fruits/total fruits) × 100 (Domínguez et al. 2012; El Chami et al. 2023). Fruit quality was assessed by measuring total soluble solids (TSS, °Brix) using a digital refractometer.

Water‐use efficiency (WUE) was calculated as total fruit yield per unit of irrigation water applied (g L^−1^), while nitrogen‐use efficiency (NUE) was calculated as total fruit yield per unit of nitrogen supplied (g g^−1^ N) (Howell 2001; Congreves et al. 2021). Total irrigation water and nitrogen supplied during the experiment were approximately 57 L plant^−1^ and 4.0 g N plant^−1^, respectively. All treatments received identical irrigation volumes and nitrogen inputs throughout the experiment via the same automated drip fertigation system; therefore, differences in WUE and NUE reflect differences in plant productivity rather than differences in resource inputs.

### 2.5 Substrate Nutrient Analysis

At the end of the experiment, substrate samples were collected from each replicate plot, homogenized, and extracted using a volumetric aqueous slurry method (Savvas and Gruda 2018). Nitrate (NO_3_^−^) concentration was determined using a Thermo Fisher Scientific ion‐selective electrode (ISE), potassium (K^+^) concentration by flame photometry (Ullah et al. 2022), and phosphate (PO_4_^3−^) concentration using a colourimetric spectrophotometric assay at 880 nm (Guo et al. 2025).

### 2.6 Statistical Analysis

Statistical analyses were performed in Jamovi (version 2.7). Growth measurements collected between Weeks 1 and 7 were analyzed using linear mixed‐effects models (GAMLj module), with AMF, NFB, week, and their interactions included as fixed effects, and block and replicate plot as random effects. Where significant main or interaction effects were detected, Bonferroni‐adjusted post hoc pairwise comparisons were performed. Final harvest measurements were analyzed using two‐way ANOVA to evaluate the main effects of AMF, NFB, and their interaction. In addition, one‐way ANOVA followed by Tukey's honestly significant difference (HSD) test was used to compare the four treatment combinations. Principal component analysis (PCA) was performed using standardized variables, and Pearson correlation analysis was used to examine relationships among measured traits. Assumptions of normality and homogeneity of variance were evaluated using residual diagnostics and Shapiro–Wilk tests.

## 3 Results

### 3.1 Number of Leaves

Nitrogen‐fixing bacteria (NFB) significantly increased leaf number (*p* < 0.001) and plant height (*p* = 0.012), whereas AMF alone had no significant effect on either parameter (*p* > 0.05). Significant AMF × NFB interactions were detected for leaf number (*p* = 0.036) and plant height (*p* = 0.022). Plants inoculated with NFB alone produced the highest leaf number (16.2 leaves), representing an increase of approximately 11.7% compared with the control (14.5 leaves), while the AMF + NFB treatment increased leaf number by 8.3%. Bonferroni‐adjusted post hoc pairwise comparisons confirmed significant differences in leaf number among treatment combinations, whereas no significant pairwise differences were detected for plant height (Table S2).

No significant treatment effects were detected for stem thickness, chlorophyll content, stomatal conductance, or flower number (*p* > 0.05; Table 1). Sampling time significantly influenced all measured traits (*p* < 0.001), reflecting normal plant growth and developmental progression throughout the experiment.

**TABLE 1:** Effects of microbial inoculation on vegetative growth, physiological traits and reproductive development of tomato plants.

Treatment	Leaf number	Plant height (cm)	Stem thickness (mm)	Chlorophyll content (SPAD)	Stomatal conductance (mmol m^−2^ s^−1^)	Flower number
Control	14.5 ± 0.49	107 ± 4.34	5.43 ± 0.12	41.1 ± 1.71	1.34 ± 0.12	6.18 ± 0.71
AMF	14.9 ± 0.46	109 ± 4.69	5.29 ± 0.11	42.5 ± 1.60	1.38 ± 0.12	6.53 ± 0.71
NFB	16.2 ± 0.55	106 ± 4.45	5.44 ± 0.11	42.2 ± 1.76	1.45 ± 0.12	5.76 ± 0.65
AMF + NFB	15.7 ± 0.58	100 ± 4.19	5.34 ± 0.11	40.6 ± 1.63	1.47 ± 0.11	5.66 ± 0.63
*p*‐value (AMF)	0.880	0.281	0.194	0.956	0.731	0.819
*p*‐value (NFB)	< 0.001	0.012*	0.742	0.777	0.324	0.245
*p*‐value (AMF × NFB)	0.036*	0.022	0.813	0.304	0.904	0.683

*Note:* Values represent means ± standard error (SE). *p*‐values were obtained from linear mixed‐effects models evaluating the effects of arbuscular mycorrhizal fungi (AMF), nitrogen‐fixing bacteria (NFB), and their interaction. Significance: *p* < 0.05 indicated by *.

### 3.2 Biomass Production and Allocation

**FIGURE 2:**
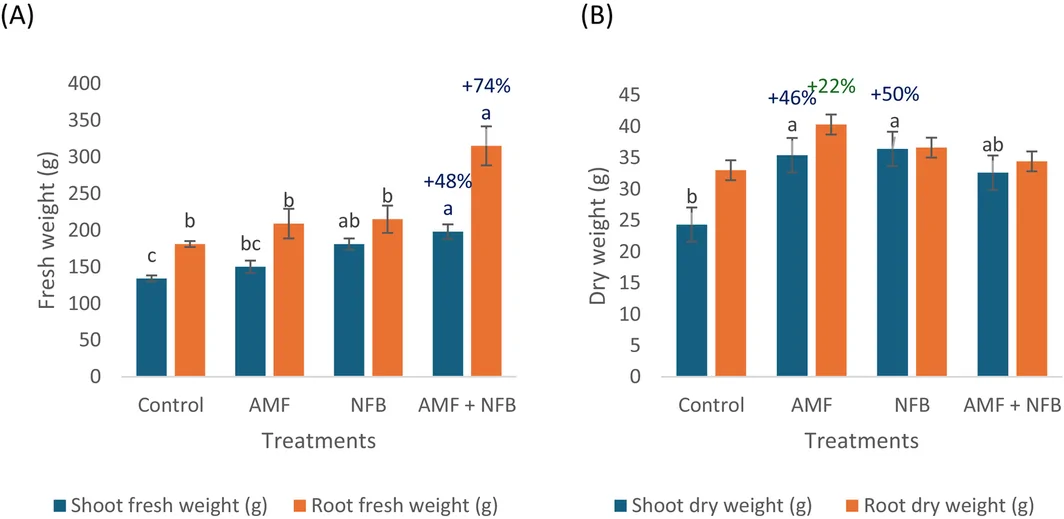
Effects of microbial inoculation on (A) shoot and root fresh weight (g) and (B) shoot and root dry weight (g) of tomato plants at final harvest. Bars represent mean values, and error bars indicate the standard error (SE) (*n* = 15). Different letters indicate significant differences among treatments according to Tukey's HSD test (*p* < 0.05). Treatments included AMF (arbuscular mycorrhizal fungi), NFB (nitrogen‐fixing bacteria), their combined application (AMF + NFB), and a noninoculated control.

Microbial inoculation significantly affected plant biomass production (Figure 2). Shoot fresh weight (SFW) was increased by AMF (*p* = 0.040) and NFB (*p* < 0.001), whereas their interaction was not significant (*p* = 0.977; Figure 2A). The largest response occurred under co‐inoculation, increasing SFW from 134.0 to 198.0 g (+48%) relative to the control. Similarly, root fresh weight (RFW) was significantly affected by AMF (*p* = 0.002) and NFB (*p* < 0.001), with co‐inoculation increasing RFW from 181.0 to 315.0 g (+74%).

Shoot dry weight (SDW) was influenced by the AMF × NFB interaction (*p* = 0.006), increasing from 24.3 g in the control to 35.4 g (+46%) and 36.4 g (+50%) in AMF‐ and NFB‐treated plants, respectively (Figure 2B). In contrast, root dry weight was not significantly affected by microbial inoculation (*p* > 0.05).

Biomass allocation and tissue dry matter responses are summarized in Table 2. The fresh shoot‐to‐root ratio was affected by the AMF × NFB interaction (*p* = 0.025), while NFB increased the dry shoot‐to‐root ratio by 51.3%, from 0.75 in the control to 1.13. Shoot dry matter percentage was influenced by the AMF × NFB interaction (*p* = 0.008), with the highest value observed in AMF‐treated plants (23.5%) and the lowest under AMF + NFB co‐inoculation (17.1%). Root dry matter percentage was affected by NFB (*p* = 0.011) and the interaction term (*p* = 0.016), ranging from 11.4% in the AMF + NFB treatment to 21.0% in the AMF treatment. Overall, NFB had the strongest effect on biomass allocation, while the interaction between AMF and NFB influenced tissue dry matter accumulation.

**TABLE 2:** Effects of AMF and NFB inoculation on biomass allocation and dry matter content of tomato plants.

Treatment	Fresh shoot: root	Dry shoot: root	Shoot dry matter (%)	Root dry matter (%)
Control	0.74 ± 0.03	0.75 ± 0.06	18.2 ± 1.6ab	18.4 ± 1.4ab
AMF	0.82 ± 0.09	0.91 ± 0.06	23.5 ± 1.29a	21.0 ± 2.5a
NFB	0.88 ± 0.06	1.13 ± 0.17	20.6 ± 1.6ab	18.1 ± 1.99ab
AMF + NFB	0.67 ± 0.05	1.14 ± 0.19	17.1 ± 1.83b	11.4 ± 1.2b
*p*‐value (AMF)	0.315	0.517	0.588	0.289
*p*‐value (NFB)	0.958	0.025*	0.218	0.011*
*p*‐value (AMF × NFB)	0.025*	0.550	0.008*	0.016*

*Note:* Values represent mean ± standard error (SE). *p*‐values were obtained from linear mixed‐effects models evaluating the effects of arbuscular mycorrhizal fungi (AMF), nitrogen‐fixing bacteria (NFB), and their interaction. Significance: *p* < 0.05 indicated by *.

### 3.3 Fruit Production, Quality and Resource‐Use Efficiency

**FIGURE 3:**
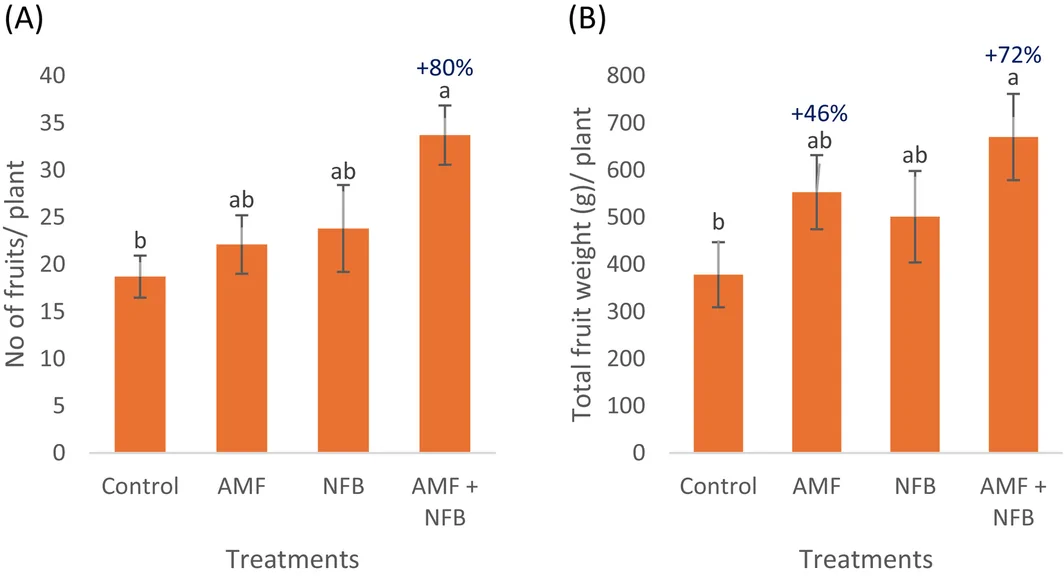
Effects of microbial inoculation on (A) total number of fruits and (B) total fruit weight (g) of tomato plants at final harvest. Bars represent mean values, and error bars indicate the standard error (SE) (*n* = 15). Different letters indicate significant differences among treatments according to Tukey's HSD test (*p* < 0.05). Treatments included AMF (arbuscular mycorrhizal fungi), NFB (nitrogen‐fixing bacteria), their combined application (AMF + NFB), and a noninoculated control.

Fruit production was significantly affected by microbial inoculation (Figure 3). Total number of fruits per plant was influenced by both AMF (*p* = 0.013) and NFB (*p* = 0.004), whereas their interaction was not significant (*p* = 0.717). The highest fruit number was observed under co‐inoculation (33.7 fruits plant^−1^), representing an 80% increase relative to the control (18.7 fruits plant^−1^). Total fruit weight per plant was significantly affected by AMF (*p* = 0.046), increasing from 378 to 553 g plant^−1^ (+46%), while the highest numerical fruit weight was recorded under co‐inoculation (670 g plant^−1^; +77%).

**FIGURE 4:**
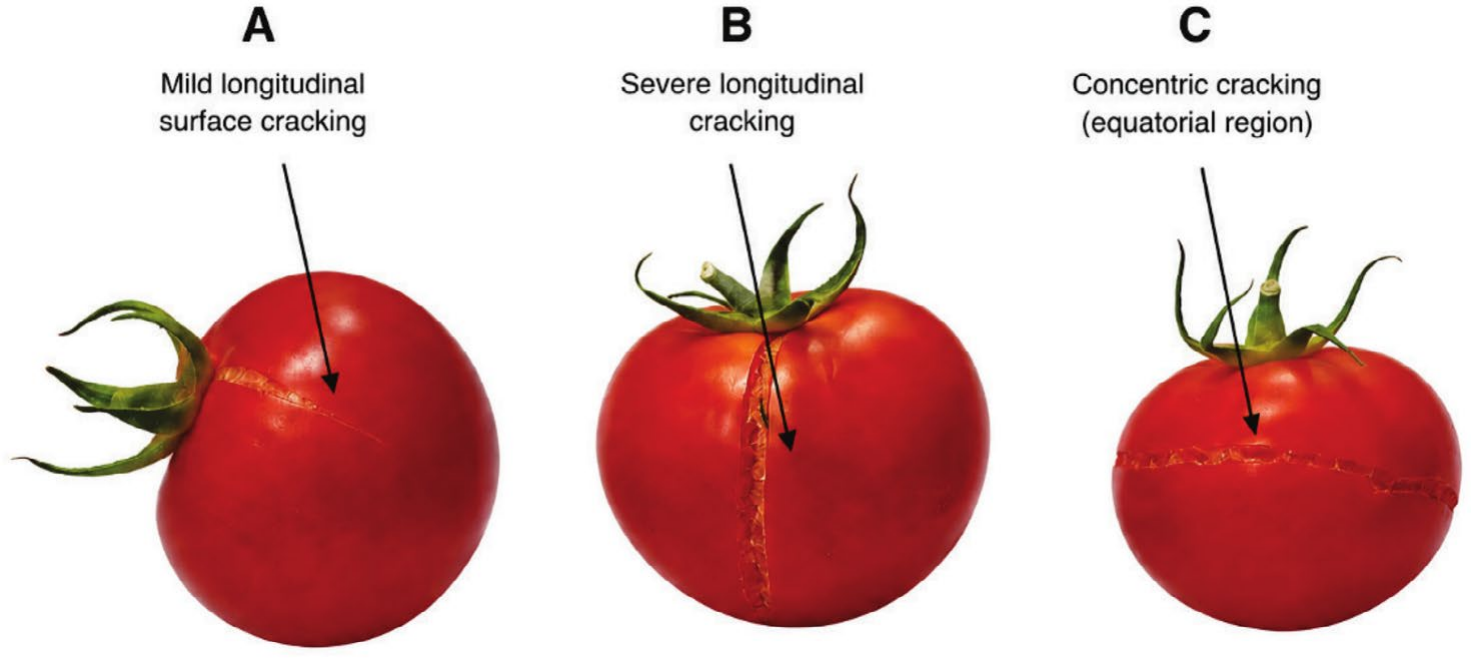
Representative tomato fruits showing cracking symptoms at harvest: (A) mild longitudinal surface cracking, (B) severe longitudinal cracking extending along the fruit axis, and (C) concentric cracking around the equatorial region. These cracking symptoms can reduce fruit quality and marketability. Photographs by the authors.

Fruit quality characteristics and resource‐use efficiency are summarized in Table 3. Marketable fruit percentage and total soluble solids were not significantly affected by microbial inoculation (*p* > 0.05). The number of unmarketable fruits was significantly affected by NFB (*p* = 0.014), whereas no significant effects of AMF or the AMF × NFB interaction was detected. Although the highest mean number of unmarketable fruits was recorded in the AMF + NFB treatment, this difference was not statistically significant. Representative examples of fruit cracking observed at harvest are shown in Figure 4. AMF had a significant positive main effect on both water‐use efficiency (WUE; *p* = 0.046) and nitrogen‐use efficiency (NUE; *p* = 0.046). Among the treatment combinations, the AMF + NFB treatment produced the highest mean WUE (11.8 g L^−1^) and NUE (168 g g^−1^ N), representing increases of approximately 78%, compared with the control.

**TABLE 3:** Effects of AMF and NFB on fruit quality and resource use efficiency.

Treatment	Unmarketable fruits (No. plant^−1^)	Marketable fruits (%)	TSS (°Brix)	WUE (g L^−1^)	NUE (g g^−1^ N)
Control	9.0 ± 12.3	42.5 ± 12.3	5.10 ± 0.62	6.6 ± 1.2	94.4 ± 17.2
AMF	14.0 ± 13.1	45.8 ± 13.1	4.92 ± 0.58	9.7 ± 1.4	138 ± 19.6
NFB	15.5 ± 14.0	50.1 ± 14.0	5.09 ± 0.64	8.8 ± 1.7	125 ± 24.2
AMF + NFB	21.1 ± 13.5	46.5 ± 13.5	5.25 ± 0.60	11.8 ± 1.61	168 ± 22.9
*p*‐value (AMF)	0.054	0.530	0.257	0.046*	0.046*
*p*‐value (NFB)	0.014*	0.146	0.952	0.162	0.162
*p*‐value (AMF × NFB)	0.921	0.484	0.311	0.970	0.970

*Note:* Values represent mean ± standard error (SE). *p*‐values were obtained from linear mixed‐effects models evaluating the effects of arbuscular mycorrhizal fungi (AMF), nitrogen‐fixing bacteria (NFB), and their interaction. Significance: *p* < 0.05 indicated by *.

Abbreviations: NUE, nitrogen use efficiency; TSS, total soluble solids; WUE, water use efficiency.

### 3.4 Principal Component Analysis

**FIGURE 5:**
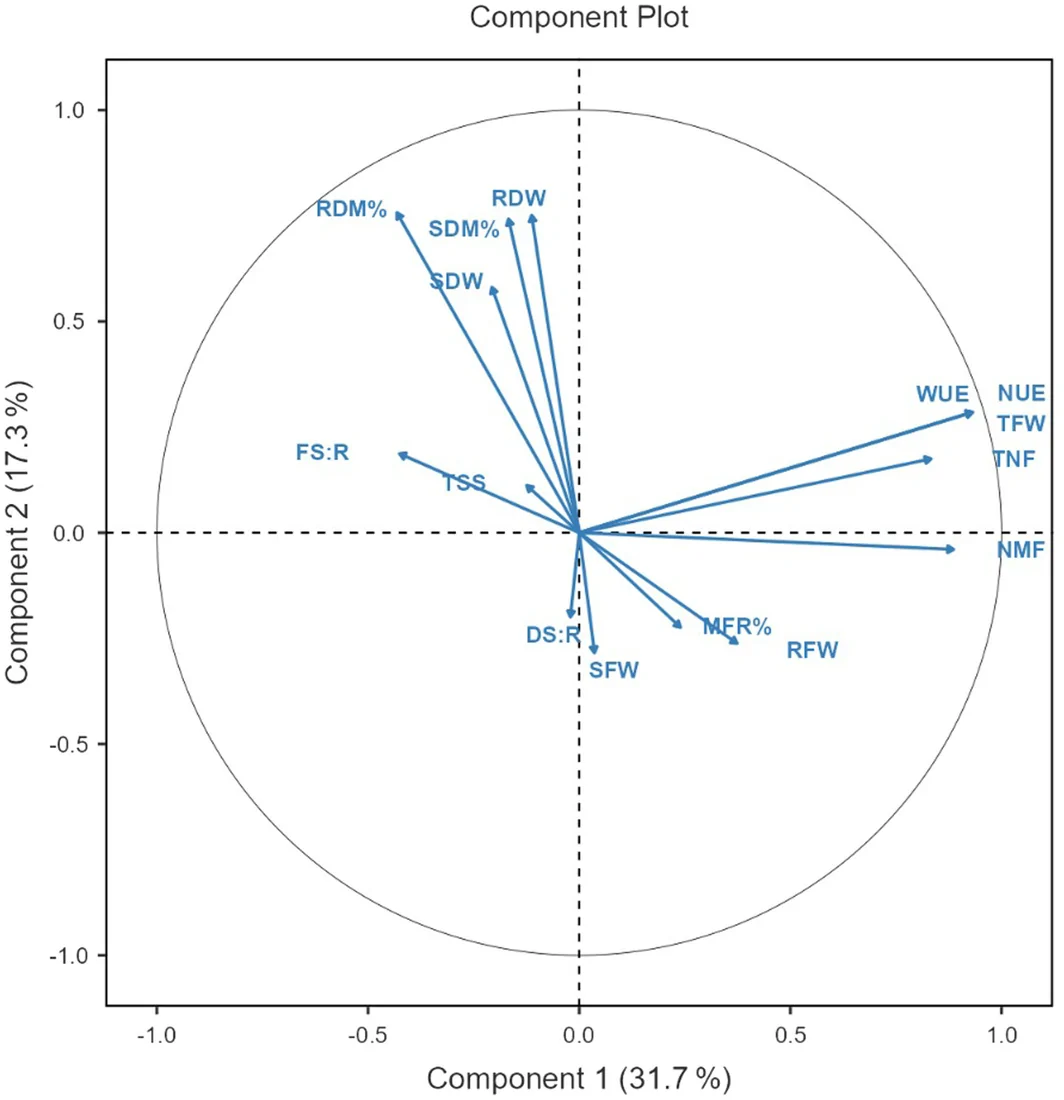
Principal component analysis (PCA) showing relationships among growth, yield, quality, and resource use efficiency traits. PC1 and PC2 explained 31.7% and 17.3% of total variance, respectively. Arrows represent variable loadings, where direction and length indicate the strength and contribution of each trait to the components. DS, R, dry shoot: Root ratio; FS, R, fresh shoot: Root ratio; MFR, number of marketable fruits; MR%, percentage of marketable fruits; NUE, nitrogen use efficiency; RDM, root dry matter; RDW, root dry weight; RFW, root fresh weight; SDM, shoot dry matter; SDW, shoot dry weight; SFW, shoot fresh weight; TFW, total fruit weight; TNF, total number of fruits; TSS, total soluble solids; WUE, water use efficiency.

Principal component analysis (PCA) explained 48.9% of the total variance across the first two components, with PC1 and PC2 accounting for 31.7% and 17.3% of the variation, respectively (Figure 5). The eigenvalues and rotated component loadings for all principal components with eigenvalues > 1 (PC1–PC5) are provided in Table S1. Variables associated with fruit productivity and resource‐use efficiency, including total fruit weight, total number of fruits, water use efficiency, and nitrogen use efficiency, showed strong positive loadings along PC1. In contrast, biomass‐related traits such as shoot dry weight, root dry weight, shoot dry matter percentage, and root dry matter percentage were more strongly associated with PC2. Traits related to biomass allocation and fruit quality, including shoot: root ratios and total soluble solids, were positioned separately from the main productivity‐related variables, indicating distinct patterns of variation among growth, yield, and quality parameters.

### 3.5 Correlation Analysis

Pearson correlation analysis revealed strong positive associations among yield‐related traits (Table 4). Total fruit weight was strongly correlated with both total fruit number (*r* = 0.80, *p* < 0.001) and marketable fruit number (*r* = 0.81, *p* < 0.001). Water‐use efficiency and nitrogen‐use efficiency were also positively correlated with fruit yield (*r* = 1.00, *p* < 0.001), reflecting their calculation from constant resource inputs.

**TABLE 4:** Pearson correlation matrix among growth, yield, and resource use efficiency traits.

Variable	SFW	RFW	FS: R	SDW	RDW	DS: R	TNF	NMF	TFW	TSS	WUE	NUE
SFW	—											
RFW	0.460***	—										
FS: R	0.268*	−0.618***	—									
SDW	0.324*	0.154	0.168	—								
RDW	0.024	0.166	−0.029	0.482***	—							
DS: R	0.288*	0.019	0.134	0.402**	−0.467***	—						
TNF	0.063	0.299*	−0.254	0.031	−0.049	0.161	—					
NMF	0.186	0.278*	−0.198	−0.143	−0.146	0.077	0.719***	—				
TFW	−0.056	0.149	−0.221	−0.104	0.052	−0.092	0.800***	0.810***	—			
TSS	−0.124	−0.171	0.091	0.049	−0.055	0.128	−0.020	−0.081	−0.065	—		
WUE	−0.056	0.149	−0.221	−0.104	0.052	−0.092	0.800***	0.810***	1.000***	−0.065	—	
NUE	−0.056	0.149	−0.221	−0.104	0.052	−0.092	0.800***	0.810***	1.000***	−0.065	1.000***	—

*Note:* Values represent correlation coefficients (r). *p* < 0.05 indicated by *, *p* < 0.01 indicated by **, and *p* < 0.001 indicated by ***.

Abbreviations: DS, R, dry shoot: Root ratio; FS, R, fresh shoot: Root ratio; NMF, number of marketable fruits; NUE, nitrogen use efficiency; RDW, root dry weight; RFW, root fresh weight; SDW, shoot dry weight; SFW, shoot fresh weight; TFW, total fruit weight; TNF, total number of fruits; TSS, total soluble solids; WUE, water use efficiency.

Biomass traits showed significant positive relationships, with shoot dry weight correlated with shoot dry matter percentage (*r* = 0.78, *p* < 0.001) and root dry weight correlated with root dry matter percentage (*r* = 0.68, *p* < 0.001). In contrast, shoot: root ratios were negatively associated with root biomass. Total soluble solids were not significantly correlated with growth, yield, or resource‐use efficiency traits (*p* > 0.05).

### 3.6 Media Nutrient Dynamics (N, P, K)

**FIGURE 6:**
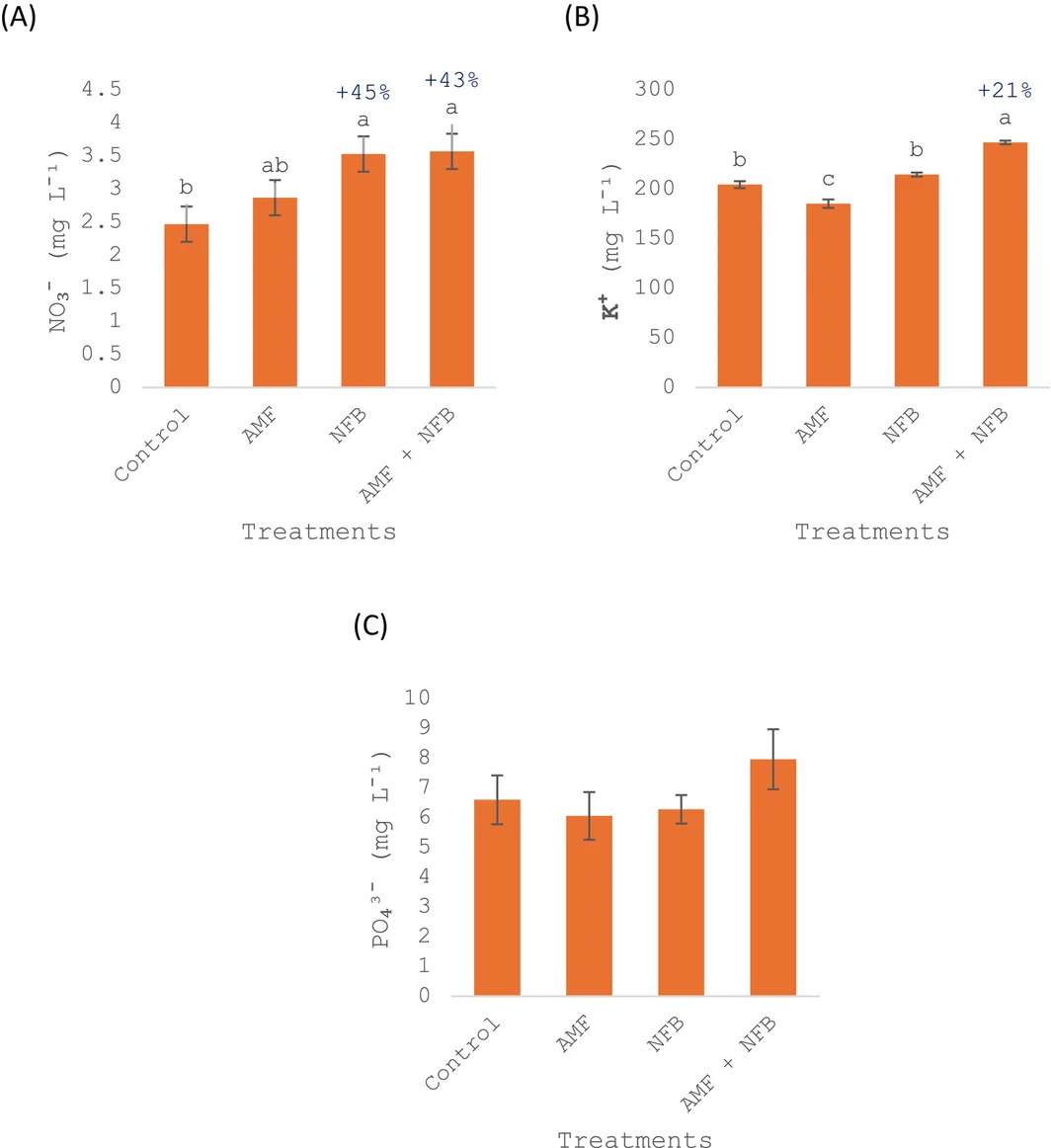
Effects of microbial inoculation on macronutrient concentrations in the growing media: (A) nitrate (NO_3_^−^, mg L^−1^), (B) potassium (K^+^, mg L^−1^), and (C) phosphate (PO_4_^3−^, mg L^−1^). Treatments include Control (no inoculation), AMF (arbuscular mycorrhizal fungi), NFB (nitrogen‐fixing bacteria), and their combined application (AMF + NFB). Bars represent mean nutrient concentrations for each treatment, and error bars indicate the standard error (SE) of the mean. Different letters above bars indicate significant differences among treatments according to Tukey's honestly significant difference (HSD) test (*p* < 0.05).

Substrate nutrient concentrations at harvest are shown in Figure 6. Nitrogen concentration was significantly influenced by treatment (*p* = 0.011). Compared with the control (2.47 mg L^−1^), Nitrate (NO_3_^−^) concentration increased by 43% in NFB‐treated plants (3.53 mg L^−1^) and by 45% under co‐inoculation (3.57 mg L^−1^), while AMF alone resulted in a smaller increase of 16% (2.87 mg L^−1^). Phosphate (PO_4_^3−^) concentration was not significantly affected by microbial inoculation (*p* > 0.05), although the highest concentration was observed under co‐inoculation (7.96 mg L^−1^). In contrast, potassium (K^+^) concentration was significantly affected by treatment (*p* < 0.001), with the AMF + NFB treatment increasing K concentration from 204.5 to 247.0 (mg L^−1^) (+21%) relative to the control.

## 4 Discussion

### 4.1 Microbial Functionality and Vegetative Growth Responses

Microbial inoculation influenced plant growth within the peat‐reduced hydroponic system, with NFB primarily enhancing vegetative development and fresh biomass production, whereas AMF was more closely associated with dry biomass accumulation and resource‐use efficiency. Increased leaf production and biomass under *A. brasilense* inoculation likely reflect improved nutrient availability and root activity during early growth stages, consistent with previous reports in tomato and other horticultural crops (Cassan et al. 2020; Ruzzi and Aroca 2015).

In contrast, AMF effects were more evident in dry biomass accumulation, suggesting enhanced nutrient acquisition and carbon assimilation through fungal hyphal networks, particularly for relatively immobile nutrients such as phosphorus (Begum et al. 2019; Abarca et al. 2024). Although co‐inoculation frequently produced the greatest numerical responses, interaction effects were generally limited, indicating complementary rather than strongly synergistic functions of the two microbial groups.

Changes in biomass allocation further suggest that microbial inoculation influenced carbon partitioning between shoots and roots. The significant effects of NFB and the AMF × NFB interaction on root dry matter percentage indicate treatment‐dependent differences in biomass composition. Although co‐inoculation substantially increased root fresh weight, the lower root dry matter percentage suggests greater root water content rather than a proportional increase in dry biomass. The relatively modest effects of AMF alone on biomass allocation traits may be related to the buffered nutrient conditions of the hydroponic system, where AMF benefits are often expressed through improved nutrient‐use efficiency rather than major architectural changes (Smith and Smith 2011).

The present findings are consistent with those of Ranasingha et al. (2026), who reported that microbial biostimulants significantly improved germination, vegetative growth and nutrient availability in ornamental kale grown in peat‐free substrate, highlighting the potential of beneficial microorganisms to enhance crop performance under reduced‐peat cultivation.

### 4.2 Reproductive Development and Resource‐Use Efficiency

Microbial inoculants exerted distinct effects on reproductive performance. Co‐inoculation produced the highest numerical fruit number, total fruit weight, water‐use efficiency (WUE), and nitrogen‐use efficiency (NUE). However, the AMF × NFB interaction was not significant, indicating that these responses reflected additive rather than synergistic effects of the two microbial inoculants. NFB primarily contributed to increased fruit number, whereas AMF had a significant positive main effect on total fruit weight, water‐use efficiency (WUE), and nitrogen‐use efficiency (NUE), indicating complementary functional roles during reproductive development. The increase in fruit number under *A. brasilense* inoculation may be associated with microbial production of phytohormones that promote flowering, fruit set, and reproductive retention (Vessey 2003; Cassan et al. 2020). However, greater fruit number did not translate into proportional increases in fruit biomass, suggesting competition among developing fruits under restricted nutrient supply. Although the AMF × NFB interaction was not significant, the highest numerical fruit weight under co‐inoculation may reflect complementary mechanisms, whereby *A. brasilense* stimulates vegetative growth and reproductive development through phytohormone production, while AMF enhances nutrient acquisition and carbon allocation to developing fruits, resulting in additive rather than synergistic effects (Rouphael et al. 2015; Santoyo et al. 2021; Othman et al. 2024).

AMF effects were more closely associated with improvements in fruit biomass accumulation and resource‐use efficiency, whereas co‐inoculation produced the greatest numerical values for these traits. Mycorrhizal fungi can enhance nutrient acquisition and influence carbon allocation to reproductive tissues, thereby supporting fruit development during later growth stages (Rouphael et al. 2015; Redoy et al. 2025). Because water and nutrient inputs were maintained constant across treatments, improved WUE and NUE reflected greater productivity per unit resource supplied rather than increased resource availability.

### 4.3 Multivariate Relationships Among Productivity and Biomass Traits

PCA and correlation analyses further supported the close association between productivity and resource‐use efficiency traits. Variables related to fruit production, including total fruit weight, fruit number, WUE, and NUE, clustered closely along PC1, indicating that microbial‐mediated improvements in productivity were associated with enhanced conversion of water and nutrient inputs into reproductive biomass. Similar relationships between yield formation and resource‐use optimisation have been reported in microbial‐assisted greenhouse systems under reduced‐input management (Niu et al. 2024; Shewangizaw et al. 2024).

Biomass‐related traits, including shoot and root dry weight and dry matter content, were more strongly associated with PC2, suggesting that structural biomass accumulation represented a distinct component of treatment variation. Positive relationships between dry biomass and dry matter percentage further indicate coordinated regulation of tissue development and carbon allocation under microbial inoculation, consistent with previous studies in horticultural crops (Pozo et al. 2015; Chandrasekaran et al. 2019).

Negative correlations between shoot ratios and root biomass suggest shifts in assimilate partitioning towards below‐ground tissues associated with nutrient acquisition and rhizosphere activity (Dellagi et al. 2020; Slimani et al. 2022). Overall, the multivariate analyses indicate that microbial inoculation enhanced productivity primarily through improved resource‐use efficiency rather than uniform stimulation of all growth traits. The first two principal components explained 48.9% of the total variance, while additional components accounted for the remaining variation (Supplementary Table S1). Accordingly, the PCA provides an exploratory summary of the relationships among the measured traits.

### 4.4 Influence of Greenhouse Microclimate on Microbial Functionality and Plant Performance

Although temperature declined and relative humidity increased during the later stages of cultivation (Figure 1B), greenhouse conditions remained within a suitable range for tomato growth (Shamshiri et al. 2018). Consistent with this, microbial inoculation had limited effects on short‐term physiological traits, including stem thickness, chlorophyll content, stomatal conductance, and flower number, whereas stronger responses were observed for biomass accumulation and productivity. Similar patterns have been reported in greenhouse tomato systems, where microbial inoculation appears to have enhanced growth and yield without consistently altering physiological parameters (Colla et al. 2017).

Treatment differences became more apparent during later growth stages, suggesting progressive establishment of microbial activity within the rhizosphere (Santoyo et al. 2021). Fruit cracking was observed in several treatments, particularly under conditions of increasing humidity during fruit development. Such responses have previously been associated with altered fruit water relations and rapid fruit expansion under protected cultivation (La Spada et al. 2024). However, the marketable fruit percentage was not significantly affected by microbial inoculation.

### 4.5 Nutrient Dynamics and Microbial Interactions in Peat‐Reduced Substrates

Microbial inoculation differentially influenced nutrient dynamics within the peat‐reduced substrate system, reflecting distinct functional roles of NFB and AMF. Increased nitrogen concentrations under NFB treatments are consistent with the capacity of *A. brasilense* to enhance nitrogen fixation and rhizosphere nutrient cycling (Bhattacharyya and Jha 2012; Vejan et al. 2016). In contrast, AMF alone produced relatively small changes in residual nutrient concentrations despite improving biomass accumulation and resource‐use efficiency, suggesting enhanced nutrient uptake and translocation into plant tissues rather than increased nutrient retention within the substrate (Smith and Smith 2011; Chiu and Paszkowski 2019).

The combined AMF + NFB treatment produced the strongest responses for nitrogen and potassium, indicating complementary microbial functionality within the rhizosphere. Similar interactions have been associated with enhanced nutrient mobilization, root exploration, and nutrient transport (Friedel and Ardakani 2021; Vejan et al. 2016). However, the limited interaction effects observed across several variables suggest largely additive rather than strongly synergistic responses, as previously reported for microbial consortia (Hart et al. 2015; Santoyo et al. 2021). Overall, the findings demonstrate that microbial inoculation can remain effective in peat‐reduced cultivation systems and may support more sustainable, low‐input horticultural production through improved nutrient availability, productivity, and resource‐use efficiency.

A limitation of this study is that AMF root colonization and *A. brasilense* establishment were not directly quantified. Consequently, although significant improvements in growth and productivity were observed following inoculation, the extent of microbial establishment within the rhizosphere cannot be confirmed. In addition, substrate nitrogen status was assessed using nitrate (NO_3_^−^) concentration only, which reflects the predominant plant‐available form of nitrogen in soilless tomato cultivation but does not account for ammonium (NH_4_^+^) or total nitrogen pools. Furthermore, the experiment was conducted over a single growing season using one tomato cultivar in a single glasshouse, which may limit the generalisability of the findings to other cultivars, seasons, and environmental conditions.

## 5 Conclusion and Future Perspectives

This study demonstrates that microbial inoculation can improve crop performance and resource‐use efficiency in peat‐reduced, low‐input horticultural systems. NFB primarily promoted vegetative growth, increasing leaf number by 11.7% and shoot fresh weight by 40%, while also contributing to greater fruit production. AMF enhanced fruit biomass, increasing total fruit weight by 46%, and exerted a significant positive main effect on WUE and NUE. Co‐inoculation produced the highest numerical responses, increasing shoot and root fresh biomass by 48% and 74%, fruit number by 80%, fruit weight by 77%, and substrate nitrate (NO_3_^−^) and potassium (K^+^) concentrations by 45% and 21%, respectively. However, these combined responses reflected complementary, additive effects rather than strong synergistic interactions between the two microbial inoculants. Importantly, these benefits were achieved under restricted water and nitrogen inputs, highlighting the potential of microbial inoculants to support more sustainable horticultural production in peat‐reduced substrates. Future research should quantify AMF root colonization and microbial establishment, while incorporating direct assessments of nutrient uptake and plant physiological processes. Multiseason and multicultivar studies are also needed to validate the broader applicability of these findings. Overall, the findings support the integration of microbial inoculants into peat‐reduced production systems as a promising strategy for improving productivity while reducing reliance on external resource inputs.

## Funding

No funding was received for this research.

## Conflicts of Interest

The authors declare no conflicts of interest.

## Supporting information


**Table S1:** Rotated component loadings, uniqueness values, eigenvalues, and percentage of variance explained for the principal component analysis (PCA). Varimax rotation was applied. Components with eigenvalues > 1 (PC1–PC5) are shown.
**Table S2:** Linear mixed‐effects model results and Bonferroni‐adjusted post hoc comparisons for repeated measurements of leaf number and plant height (Weeks 1–7).

## Data Availability

The data that support the findings of this study are available in Figshare at https://figshare.com/s/b1d4896e789b325699ab.
