# Supplementary material for: Arbuscular Mycorrhizal Fungi and Azospirillum brasilense Enhance Growth and Resource Use Efficiency of Tomato Under Resource‐Limited Peat‐Reduced Cultivation
Source: Plant Environ Interact. 2026 Aug 23;7(4):e70205. doi: 10.1002/pei3.70205 (PMC13500900; doi:10.1002/pei3.70205)
Supplement: Supplementary file 1 [file PEI3-7-e70205-s001.docx]

| **Variable** | **PC1** | | **PC2** | | **PC3** | | **PC4** | **PC5** | | **Uniqueness** |
| --- | --- | --- | --- | --- | --- | --- | --- | --- | --- | --- |
| SFW |  | |  | |  | | 0.305 | 0.858 | | 0.143 |
| RFW |  | |  | | -0.802 | |  | 0.481 | | 0.089 |
| FS: R |  | |  | | 0.926 | |  |  | | 0.059 |
| SDW |  | | 0.954 | |  | |  |  | | 0.030 |
| RDW |  | | 0.646 | |  | | -0.713 |  | | 0.063 |
| DS: R |  | |  | |  | | 0.914 |  | | 0.093 |
| SDM |  | | 0.854 | |  | |  | -0.396 | | 0.113 |
| RDM |  | | 0.418 | | 0.659 | | -0.567 |  | | 0.036 |
| TNF | 0.841 | |  | |  | |  |  | | 0.192 |
| NMF | 0.847 | |  | |  | |  | 0.310 | | 0.141 |
| MFR% |  | |  | |  | |  | 0.652 | | 0.519 |
| TFW | 0.983 | |  | |  | |  |  | | 0.021 |
| TSS |  | |  | |  | |  | -0.352 | | 0.773 |
| WUE | 0.983 | |  | |  | |  |  | | 0.021 |
| NUE | 0.983 | |  | |  | |  |  | | 0.021 |
| **Component** | | **Eigenvalue** | | **Variance (%)** | | **Cumulative (%)** | | |  |  |
| PC1 | | 4.748 | | 31.7 | | 31.7 | | |  |  |
| PC2 | | 2.589 | | 17.3 | | 48.9 | | |  |  |
| PC3 | | 2.086 | | 13.9 | | 62.8 | | |  |  |
| PC4 | | 1.711 | | 11.4 | | 74.2 | | |  |  |
| PC5 | | 1.550 | | 10.3 | | 84.6 | | |  |  |

**Supplementary Table S1.** Rotated component loadings, uniqueness values, eigenvalues, and percentage of variance explained for the principal component analysis (PCA). Varimax rotation was applied. Components with eigenvalues >1 (PC1–PC5) are shown.

Treatments included AMF (arbuscular mycorrhizal fungi), NFB (nitrogen-fixing bacteria), their combined application (AMF + NFB), and a non-inoculated control. SFW = shoot fresh weight; RFW = root fresh weight; SDW = shoot dry weight; RDW = root dry weight; FS:R = fresh shoot: root ratio; DS:R = dry shoot: root ratio; SDM = shoot dry matter; RDM = root dry matter; TNF = total number of fruits; MFR = number of marketable fruits; MR% = percentage of marketable fruits; TFW = total fruit weight; TSS = total soluble solids; WUE = water use efficiency; NUE = nitrogen use efficiency.

**Supplementary Table S2. Linear mixed-effects model results and Bonferroni-adjusted post hoc comparisons for repeated measurements of leaf number and plant height (Weeks 1–7).**

**A. Linear mixed-effects model results**

| **Variable** | **Effect** | **F** | **df** | **Residual df** | **p-value** |
| --- | --- | --- | --- | --- | --- |
| **Leaf number** | AMF | 0.023 | 1 | 414 | 0.881 |
|  | NFB | 42.449 | 1 | 414 | <0.001* |
|  | Block | 0.608 | 1 | 414 | 0.436 |
|  | Week | 2721.974 | 1 | 414 | <0.001* |
|  | AMF × NFB | 4.431 | 1 | 414 | 0.036* |
| **Plant height** | AMF | 1.164 | 1 | 412 | 0.281 |
|  | NFB | 6.398 | 1 | 412 | 0.012* |
|  | Block | 0.190 | 1 | 412 | 0.663 |
|  | Week | 2002.440 | 1 | 412 | <0.001* |
|  | AMF × NFB | 5.245 | 1 | 412 | 0.023* |

**B. Bonferroni-adjusted post hoc comparisons**

**Leaf number**

| **Comparison** | **Mean difference** | **SE** | **t** | **p-value** |
| --- | --- | --- | --- | --- |
| AMF + NFB vs AMF | 0.838 | 0.269 | 3.12 | 0.012* |
| AMF + NFB vs NFB | -0.429 | 0.269 | -1.59 | 0.669 |
| AMF + NFB vs Control | 1.210 | 0.269 | 4.50 | <0.001* |
| AMF vs NFB | -1.267 | 0.269 | -4.71 | <0.001* |
| AMF vs Control | 0.371 | 0.269 | 1.38 | 1.000 |
| NFB vs Control | 1.638 | 0.269 | 6.10 | <0.001* |

**Plant height**

| **Comparison** | **Mean difference** | **SE** |  | **t** | **p-value** |
| --- | --- | --- | --- | --- | --- |
| AMF + NFB vs AMF | 8.83 | 2.59 |  | 3.41 | 0.492 |
| AMF + NFB vs NFB | -6.17 | 2.59 |  | -2.38 | 0.757 |
| AMF + NFB vs Control | -6.61 | 2.59 |  | -2.55 | 0.715 |
| AMF vs NFB | 2.66 | 2.59 |  | 1.03 | 0.974 |
| AMF vs Control | 2.22 | 2.59 |  | 0.86 | 0.985 |
| NFB vs Control | 0.44 | 2.59 |  | 0.17 | 1.000 |

*Values are based on linear mixed-effects models fitted to repeated measurements collected from Weeks 1–7. Pairwise comparisons were adjusted using the Bonferroni method. Significant differences are indicated by * (p < 0.05). Treatments included AMF (arbuscular mycorrhizal fungi), NFB (nitrogen-fixing bacteria), their combined application (AMF + NFB), and a non-inoculated control.*
